# Current News and Opinion

**Published:** 1885-10

**Authors:** 


### Current News and Opinion.

### ABOUT EXPOSED DENTAL PULPS.

Recently I received a professional call from one of the oldest and most reputable members of our fraternity, who was suffering somewhat from a slight attack of pulpitis in an inferior molar. A fair sized cavity on its posterior approximal surface extended to the pulp chamber; a small point of the pulp was exposed, and the tooth was slightly sore to the touch.

After due examination I asked my friend if he wished the tooth treated with a view of saving the pulp, or would he prefer to have it devitalized and removed? "If not devitalized, I fear after-trouble," was the reply, "for although I have been a strong advocate for saving exposed pulps, and have met with some undoubted successes, yet many times where I had taken all possible pains to save them, and had flattered myself that all was right, I have afterwards had patients whose teeth I had thus treated come back to me in an agonized condition, to have the fillings removed and the teeth again treated—in the end to have the pulps removed and the root canals filled." "And has not this been the experience of most of us?" I remarked. "Undoubtedly," was the reply.

Let us all be candid and honest in this matter, and not claim credit for greater successes than we deserve, or actually gain. That, under favorable circumstances, recently exposed pulps are frequently saved, is a fact that few, if any, will deny; but where pulps are sickly, or partly congested, the chance of restoring them to health and insuring lasting peace within the pulp chamber, is a matter of very grave doubt. C. E. F.

### DENTAL EDUCATION IN GREAT BRITAIN.

The September number of the *Dental Record*, of London, England, is given up mainly to information concerning dental education in Great Britain—"The Student's Number." The qualifying degree is that of L. D. S. (Licentiate of Dental Surgery), and this may be obtained from a number of different sources. The following is condensed from *The Record:*

The Professional Education of the Dental Student consists of Apprenticeship, or instruction in Mechanical Dentistry, for a period of not less than three years: Attendance on Lectures, &c., at a General Hospital and Medical School for two Winter and one Summer Sessions—eighteen months: Also attendance at a Dental Hospital and School for two years. The attendance at the General Medical and at the Special Dental Hospitals and Schools may be carried out simultaneously, and completed in two years. This plan of work takes up the whole of the Student's time, and it is impossible for him to concurrently engage in any Mechanical or other employment.

Before commencing his Professional Education, the Dental Student must pass a Preliminary Examination in Arts. The examination most to be recommended is the matriculation of the University of London. Passing that examination will enable the student subsequently to present himself for an University degree, should he desire to do so.

Any one who commenced his professional education before the 22nd July, 1878, is exempt from the Preliminary Examination.

After passing a Preliminary Examination, the student must receive at least three years' instruction in Mechanical Dentistry under a registered Dentist. It should be distinctly understood that the Royal College of Surgeons of England consider those three years of instruction, whether in the form of serving articles, or apprenticeship to Mechanical Dentistry, or otherwise, as Professional Education; therefore, they follow the Preliminary Examination.

Having received a certificate of his Preliminary Examination, and commenced his Professional Education, either by apprenticeship or by hospital studies, it is necessary to register the same at the Medical Council Office, 299 Oxford Street, London. This must be done within fifteen days from the commencement of the pupil's professional studies. The beginning of such studies will not be recognized by any of the qualifying bodies as dating earlier than fifteen days before the time of registration.

Any one registered as a Medical Student must also register as a Dental Student, if he be such.

Having served his articles, the student may enter a General or a Dental Hospital, or both, and complete the Curriculum of at least four years of study from the date of registration; after which he is eligible to be admitted to examination for the Dental License.

Though the possession of the License in Dental Surgery is necessary for a name to be entered on the *Dentists' Register*, the student is strongly recommended to obtain some additional qualification in either Medicine or Surgery. On the other hand, though a Medical or a Surgical, other than the special Dental, qualification entitles its possessor to practice Dentistry, yet the course of study for such does not include any Dental instruction. Therefore, to be a Dentist requires a more or less complete compliance with the Dental Curriculum.

Candidates for a diploma in dental surgery must produce certificates of having been engaged during four years in professional studies, and of having received three years' instruction in mechanical dentistry from a registered practitioner.

The preliminary examination includes the following:

1. English Language, including Grammar and Composition.
2. Latin, including Grammar, Translation from specified authors, and Translation of easy passages not taken from such authors.

3. Elements of Mathematics, comprising (*a*) Arithmetic, including Vulgar and Decimal Fractions; (*b*) Algebra, including Simple Equations; (*c*, Geometry, including the first book of Euclid, with easy questions on the subject-matter of the same.

4. Elementary Mechanics of Solids and Fluids, comprising the Elements of Statics, Dynamics, and Hydrostatics.

5. One of the following optional subjects:—(*a*) Greek, (*b*) French, (*c*) German, (*d*) Italian, (*e*) any other Modern Language, (*f*) Logic, (*g*) Botany, (*h*) Zoology, (*i*) Elementary Chemistry.

The examining bodies, to grant preliminary certificates, are:

The Universities of Oxford, Cambridge, Durham, London, Edinburgh, Aberdeen, Glasgow, St. Andrew's, Dublin, Victoria University; Queen's University, of Ireland; Royal University of Ireland; Oxford and Cambridge Schools' Examination Board, with some medical and other boards and societies, and certain Indian, Colonial, and Foreign Universities and Colleges.

The examination for the Diploma in Dental Surgery by the Royal College of Surgeons of England demands the following certificates:

1. Of being twenty-one years of age.

2. Of having been engaged during four years in the acquirement of professional knowledge.

3. Of having attended, at a school or schools recognized by this College, not less than one of each of the following courses of lectures, delivered by lecturers recognized by this College, namely: Anatomy, Physiology, Surgery, Medicine, Chemistry and Materia Medica.

4. Of having attended a second winter course of lectures on Anatomy, or a course of not less than twenty lectures on the Anatomy of the Head and Neck, delivered by lecturers recognized by this College.

5. Of having performed dissections at a recognized school during not less than nine months.

6. Of having completed a course of chemical manipulation, under the superintendence of a teacher or lecturer recognized by this College.

7. Of having attended, at a recognized Hospital or Hospitals in the United Kingdom, the practice of Surgery and Clinical Lectures on Surgery during two winter sessions.

8. Of having attended, at a recognized school, two courses of lectures upon each of the following subjects, namely:—Dental Anatomy and Physiology (Human and Comparative), Dental Surgery, Dental Mechanics, and one course of lectures on Metallurgy, by lecturers recognized by this College.

9. Of having been engaged during a period of not less than three years in acquiring a practical familiarity with the details of Mechanical Dentistry, under the instruction of a competent Practitioner. In the cases of qualified Surgeons, evidence of a period of not less than two, instead of three years, of such instruction will be sufficient.

10. Of having attended at a recognized Dental Hospital, or in the Dental Department of a recognized General Hospital, the practice of Dental Surgery during the period of two years.

The examination by the different legally qualified bodies varies considerably, and hence the diploma of one body may be more valuable than that of another. The institutions qualified to grant the dental degrees are :

The Royal College of Surgeons, of England.
The Royal College of Surgeons, of Edinburgh.
The Faculty of Physicians and Surgeons, of Glasgow.
The Royal College of Surgeons, of Ireland.

The special Dental Schools are :

The Dental Hospital, of London.
National Dental Hospital, London.
Owen's College, and Victoria Dental Hospital, Manchester.
Queen's College, Birmingham, and Birmingham Dental Hospital.
University College, Liverpool, and Liverpool Dental Hospital.
Dental Hospital, of Exeter.
Edinburgh Dental Hospital.
Anderson's College, Dental Hospital and School, Glasgow.
Dental Hospital, of Dublin.

In addition, the following general Medical Hospitals, of London, receive dental students :

Bartholomew's Hospital; Charing Cross Hospital; Guy's Hospital; London Hospital; Middlesex Hospital; St. George's Hospital; St. Mary's Hospital; St. Thomas' Hospital; Westminster Hospital.

Candidates who were in practice before the first day of August, 1878, or those not in practice, but who had commenced their apprenticeship as dentists before the first day of August, 1875, and who are unable to furnish the Board of Examiners with the certificates of lectures and hospital attendance required by the regulations, may be admitted to examination *sine curriculo.*

### CHANGE OF LOCATION.

The editorial office of the INDEPENDENT PRACTITIONER has been removed from No. 11 West Chippewa Street, to No. 208 Franklin Street. The distance is not great—only about a block—but the editor will find himself in much more pleasant quarters, in his own house, where he will be glad to welcome his personal friends and those of the journal. All editorial communications should henceforth be addressed to the new number.

### CONSOLIDATION.

The *Medical Chronicle*, of Baltimore, is consolidated with the *Philadelphia Medical Times*, Dr. Rohé, the editor of the former, becoming the associate editor of the latter. He has proved himself such an accomplished writer that it would have been a serious loss to medical journalism had he retired from the field altogether.

### CONNECTICUT VALLEY DENTAL SOCIETY.

The twenty-second annual meeting of the Connecticut Valley Dental Society will be held at Springfield, Mass., Thursday and Friday, November 5th and 6th.

The programme, as already arranged, includes a number of interesting papers and reports, indicating that this will be one of the instructive meetings of the year.

Those having the *blanks* which this society sent out last January are requested to *return them at once*, that a report from them may be presented at this meeting. GEO. A. MAXFIELD,
Secretary.

---

### SEVENTH AND EIGHTH DISTRICT DENTAL SOCIETIES.

A union meeting of the Seventh and Eighth District Dental Societies of the State of New York will be held at the Genesee House, in Buffalo, October 27th and 28th, 1885. An excellent programme will be presented, including addresses by eminent men outside of dentistry, upon subjects cognate to dental practice, the exhibition of histological and pathological preparations by means of the stereopticon, etc. It is proposed to make the meeting especially one of reunion of the older dentists of Western New York. All are cordially invited to be present.

---

### FIFTH AND SIXTH DISTRICT DENTAL SOCIETIES.

A union meeting of the Fifth and Sixth District Dental Societies, of the State of New York, will be held in the Supervisors' room, at Binghamton, on Tuesday and Wednesday, Oct. 13th and 14th, 1885. All members of the profession are cordially invited to be present. C. J. PETERS, D. D. S.,
Sec'y Fifth Dist. Dent. Soc'y.
E. D. DOWNS, D. D. S.,
Sec'y Sixth Dist. Dent. Soc'y.

---

### THE OHIO STATE DENTAL SOCIETY.

The first annual meeting of the Ohio State Dental Society (reorganized) will be held in Chillicothe, commencing on the last Wednesday of October, 1885. J. R. CALLAHAN,
Secretary.

